# Transcriptome expression profile of compound-K-enriched red ginseng extract (DDK-401) in Korean volunteers and its apoptotic properties

**DOI:** 10.3389/fphar.2022.999192

**Published:** 2022-12-01

**Authors:** Jong Chan Ahn, Ramya Mathiyalagan, Jinnatun Nahar, Zelika Mega Ramadhania, Byoung Man Kong, Dong-Wook Lee, Sung Keun Choi, Chang Soon Lee, Vinothini Boopathi, Dong Uk Yang, Bo Yeon Kim, Hyon Park, Deok Chun Yang, Se Chan Kang

**Affiliations:** ^1^ Graduate School of Biotechnology, College of Life Sciences, Kyung Hee University, Yongin-si, South Korea; ^2^ Department of Oriental Medicinal Biotechnology, College of Life Science, Kyung Hee University, Yongin-si, South Korea; ^3^ Hanbangbio Inc., Yongin-si, South Korea; ^4^ Daedong Korea Ginseng Co., Ltd., Geumsan-gun, South Korea; ^5^ Exercise Nutrition & Biochemistry Lab, Kyung Hee University, Yongin-si, South Korea

**Keywords:** fermented red ginseng, compound K (CK), pharmacokinetics, clinical trial, mRNA sequence, antioxidant, cancer, inflammation

## Abstract

Ginseng and ginsenosides have been reported to have various pharmacological effects, but their efficacies depend on intestinal absorption. Compound K (CK) is gaining prominence for its biological and pharmaceutical properties. In this study, CK-enriched fermented red ginseng extract (DDK-401) was prepared by enzymatic reactions. To examine its pharmacokinetics, a randomized, single-dose, two-sequence, crossover study was performed with eleven healthy Korean male and female volunteers. The volunteers were assigned to take a single oral dose of one of two extracts, DDK-401 or common red ginseng extract (DDK-204), during the initial period. After a 7-day washout, they received the other extract. The pharmacokinetics of DDK-401 showed that its maximum plasma concentration (Cmax) occurred at 184.8 ± 39.64 ng/mL, Tmax was at 2.4 h, and AUC_0–12h_ was 920.3 ± 194.70 ng h/mL, which were all better than those of DDK-204. The maximum CK absorption in the female volunteers was higher than that in the male volunteers. The differentially expressed genes from the male and female groups were subjected to a KEGG pathway analysis, which showed results in the cell death pathway, such as apoptosis and necroptosis. In cytotoxicity tests, DDK-401 and DDK-204 were not particularly toxic to normal (HaCaT) cells, but at a concentration of 250 μg/mL, DDK-401 had a much higher toxicity to human lung cancer (A549) cells than DDK-204. DDK-401 also showed a stronger antioxidant capacity than DDK-204 in both the DPPH and potassium ferricyanide reducing power assays. DDK-401 reduced the reactive oxygen species production in HaCaT cells with induced oxidative stress and led to apoptosis in the A549 cells. In the mRNA sequence analysis, a signaling pathway with selected marker genes was assessed by RT-PCR. In the HaCaT cells, DDK-401 and DDK-204 did not regulate FOXO3, TLR4, MMP-9, or p38 expression; however, in the A549 cells, DDK-401 downregulated the expressions of MMP9 and TLR4 as well as upregulated the expressions of the p38 and caspase-8 genes compared to DDK-204. These results suggest that DDK-401 could act as a molecular switch for these two cellular processes in response to cell damage signaling and that it could be a potential candidate for further evaluations in health promotion studies.

## Introduction

**FIGURE 1:**
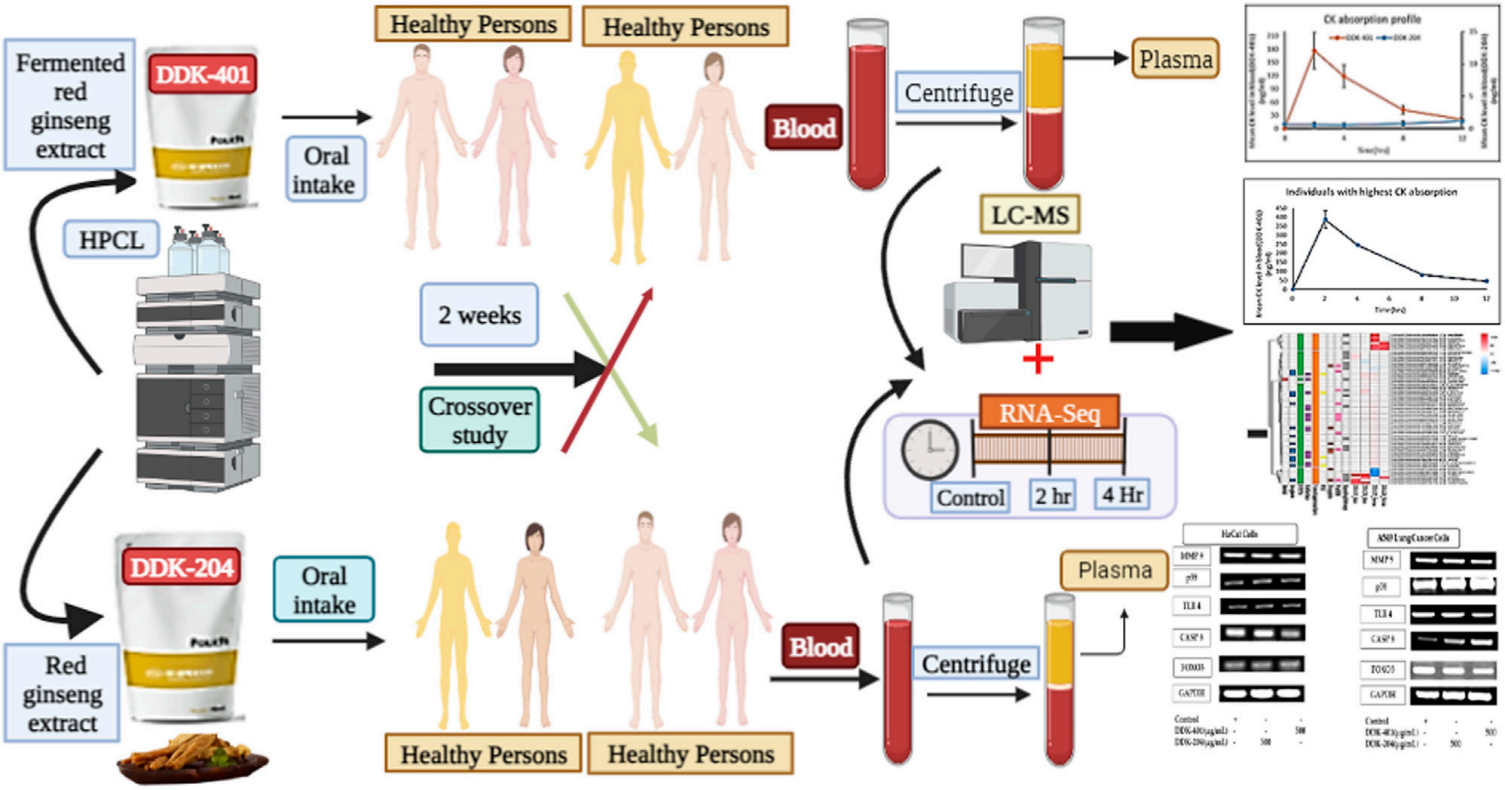
Graphical illustration of the fermented red ginseng extract (DDK-401) and red ginseng extract (DDK-204) analyzed by Korean volunteers.

Traditional Chinese medicine (TCM) is the oldest medicinal practice in history, and its basic rule is to incorporate the principles of Yin and Yang in all of its therapeutics. Various other medical systems, particularly oriental medicine rooted in Chinese medicine, are still considered valid (Borman and Kim, 1966). Given that ginseng is mentioned as a medicinal herb in the Classic Herbal of Shennong, which was written around 100 CE, it is apparent that the therapeutic history of ginseng began in the ancient times (Dharmananda, 2002). Ginseng is a common name for plants in the Panax family. In Chinese, “gin” refers to man, and “seng” means essence; it was known as a gift to man from the deity of the mountains in ancient times. It is also known as a plant made of crystals of the essence needed to cure human diseases (Hu, 1976). In addition to historical references, fossil evidence shows that plants from the Araliaceous family existed 65 million years ago, and the Panax species are about 38 million years old (Court, 2000). The book of Shanghan Lun, written in 220 CE, mentions the medical applications of and methods to measure 107 formulas, of which 21 contain ginseng. Even today, most people practicing TCM follow the formulations of Shanghan Lun. Dharmananda (2002) documented the medical history of ginseng from 220 CE to the 20th century. Experts in various fields, such as oncology (Majeed et al., 2018; Nakhjavani et al., 2019; Yu-hang et al., 2019), central nervous system (Radad et al., 2011), energy metabolism (Zhang et al., 2017), stroke (Liu et al., 2019), depression (Jin et al., 2019), infectious diseases (Nguyen and Nguyen, 2019), neurology (Huang et al., 2019), skin disorders (Kim and Kim, 2018), Parkinson’s disease (González-Burgos et al., 2015), autophagy (Wu et al., 2019), inflammation (Ramadhania et al., 2022), diabetes (Zhou et al., 2019), hepatology (Gao et al., 2017), obesity (Li and Ji, 2018), mitochondrial activity (Zhou et al., 2019c), cardiology (Zheng et al., 2012), antimicrobials (Kachur and Suntres, 2016), immune functions (Kang and Min, 2012; Riaz et al., 2019), and molecular signaling pathways (Mohanan et al., 2018), have reviewed the continuous details of ginseng’s efficacy to understand how the ginsenosides disrupt diseases as well as their related mechanisms. Ginseng is generally classified into white, red, and black ginseng according to different stages of processing, and all of these ginseng products are available in the market. Depending on the stage of processing, the therapeutic metabolite content varies widely. The different therapeutic functions attributed to products from different steps have been classified previously (Jin et al., 2015; Shin et al., 2019; Zhu et al., 2019). The dried fresh roots are called white ginseng. The process of obtaining red ginseng begins by washing the fresh ginseng roots in water to remove soil particles; then, they are steamed at 90–98 °C for 1–3 h. This process is repeated once or twice more to achieve appropriate gelatinization of the ginseng starch; the product is then dried until the root has a moisture content of 15–18%. This processing method has been used since 1123 CE, although it has been optimized in various ways (Lee et al., 2015). The value of the resulting formulation depends upon the key chemical ingredient, i.e., tri-terpenoid saponins called ginsenosides, which are the key metabolites of ginseng (Christensen, 2008; Liu, 2012; Boopathi et al., 2020). Ginsenosides are classified into major and minor based on their molecular weights. Naturally biosynthesized ginsenosides in plants are called major ginsenosides, and the converted forms are called minor ginsenosides. The conversion method involves hydrolysis of the glycose molecules in the backbone moiety using physical (heat, microwave, and puffing), chemical (acid and alkali), or enzymatic (various glycosidase enzymes, genetic engineering, lactic acid bacteria) techniques. The bioavailability of the major and minor ginsenosides are the key issue in promoting ginsenosides as drug candidates. Ginsenoside compound K (CK) is one of the major metabolites that reaches systemic circulation, where it has its various pharmacological effects (Sharma and Lee, 2020; Murugesan et al., 2022). Recently, the benefits of fermented functional food products (i.e., probiotics) that enhance human gut health and immunity have gained attention for their potential in treating various chronic diseases. The ginseng functional food industry has also risen to leverage the efficacy of ginseng. Another current trend focuses on the advantages of drug combinations over individual drugs, with primarily enhanced efficacy in slowing or reversing disease progression and reduced side effects (Liu et al., 2020). However, choosing effective combinations through trial and error is both tedious and expensive. Therefore, a principle similar to that long used in TCM is suggested to moderate the various side effects without requiring systematic evaluations of the extract formulations (Posadzki et al., 2013). However, such a system needs to accommodate modern medicine by offering valid evidence when identifying novel drug combinations practically (Shin et al., 2021). Therefore, various studies have been conducted to assess the therapeutic effects of individual ginsenosides as well as crude, red, or fermented red ginseng extracts in animal models and human trials (Lee et al., 2012; Jung et al., 2013; Kim, 2013; Sohn et al., 2013; Choi et al., 2016; Choi et al., 2018; Ban et al., 2021; Panossian et al., 2021), but these datasets are insufficient to conclusively demonstrate the effectiveness of ginseng/ginsenosides at the molecular level. In the present study, we examine a CK-enriched fermented ginseng extract DDK-401 in a human trial with a healthy population to understand its effects on various signaling pathways (Figure 1). Moreover, we elucidate its effects on the functional and therapeutic markers already approved for treating various diseases.

## Materials and methods

### Ethical committee and study design

A randomized, open-label, single-dose, two-period, two-sequence, crossover study was performed with healthy Korean male and female subjects. This study was performed in accordance with the principles of the Declaration of Helsinki and Korean Good Clinical Practice guidelines. Informed written consent was obtained from each subject in advance. The study was approved by the Institutional Review Board of Kyung Hee University Hospital (KHGIRB-21-419). Eleven healthy male and female Koreans were enrolled in this study, and their age details are shown in Supplementary Table S1. We expected to find large individual variability in the pharmacokinetic profile of CK. To reduce the individual variability in pharmacokinetics caused by sex, we combined and calculated the means of the pharmacokinetic data from the male and female groups separately. The exclusion criteria were any significant clinical illness within 2 weeks before the study, i.e., history of high blood pressure, diabetes, and cardiovascular, hepatic, renal, hematological, gastrointestinal, neurologic, or psychiatric disease; blood donation within 8 weeks before the study; and use of any medications, including prescription and over-the-counter drugs, within 2 weeks before the study. In addition, subjects who previously experienced adverse reactions to ginseng were excluded.

The enrolled subjects were assigned to receive a single oral dose of one of two extracts, DDK-401 (100 mL spout pouch, combination of well-known representative ginsenosides Rg1, Rb1, and Rg3 at 21.51 mg and ginsenoside CK at 31.19 mg) or DDK-204 (100 mL spout pouch, ginsenosides Rg1, Rb1, and Rg3 at 11.29 mg and ginsenoside CK at 0 mg) during the first period. After a 7-day washout, each subject received the other extract. The dose for oral administration was chosen based on the recommended total daily intake of each investigational product.

### DDK-401 and DDK-204 extract preparations

CK-enriched fermented ginseng extract (DDK-401) and common red ginseng extract (DDK-204) were supplied by Deadong Korea Ginseng Co., Ltd. (Geumsan, Korea). First, the red ginseng powder was dissolved in a mixture of water and food-grade alcohol and extracted at 75 ± 5 °C. This extraction procedure was repeated 4 times. Then, the supernatant was collected and evaporated at 60 ± 5 °C with 500–760 mmHg vacuum until the sugar content was 65 brix and solid content was ≥60%. Finally, the sample was sterilized at 80–85 °C for 30–40 min and aged at 60 ± 5 °C for 24–75 h; this product was named DDK-204 and used as the control.

Second, the red ginseng concentrate (60 brix) was diluted in water until the solid content was 5%. Then, an enzyme mixture (pectinase and *β*-glucosidase) was added to the diluted red ginseng concentrate at a concentration of 3% and reacted at 60 ± 2 °C for 114–168 h at 3000 rpm. Thereafter, the enzyme was inactivated at 90 °C for 30 min. Next, the sample was evaporated at 55 ± 5 °C and 500–760 mmHg vacuum until the solid content reached 40%. Then the sample was dissolved in 80% food-grade alcohol and incubated for 1–2 h. Following this, centrifugation was performed at 0.5 m^3^/h for 5–6 h, and the supernatant was collected. A second evaporation was then performed at 55 ± 5 °C and 500–760 mmHg vacuum until the sugar content was 65 brix and solid content was ≥60%. The concentrate was then fermented with a mixture of *Lactobacillus* species at 1% concentration and 37 °C for one day. The resulting CK-enriched red ginseng concentrate was named DDK-401 and stored in the refrigerator until it was used for the analysis and bioassays.

### Chromatographic conditions for analyzing the ginsenoside profiles of DDK-401 and DDK-204

One g each of DDK-401 and DDK-204 were dissolved in 50 mL of 70% methanol and filtered with a 0.45 μm membrane filter. The samples were then injected into an Ultimate 3000 HPLC system with a PRONTOSIL 120-5-C18 ACE-EPS (250 × 4.6 mm i.d., 5 µm particle size) (Bischoff Chromatography, Leonberg, Germany). The mobile phase consisted of water (solvent A) and acetonitrile (solvent B) in the following gradients: 0–10 min, 20% B; 10–42 min, 29% B; 42–67 min, 41% B; 67–70 min, 47% B; 70–90 min, 71% B; 90–95 min, 71% B. The flow rate of the mobile phase was 1.0 mL/min, and an injection volume of 10 µL was used in the quantitative analysis. The column temperature was maintained constant at 40 °C. The ginsenoside profiles were determined at 203 nm.

#### Preparation of standard solution for quantitative calibration

A standard stock solution of 10% was prepared by dissolving accurately weighed quantities of the standard for each ginsenoside in high-performance liquid chromatography (HPLC)-grade methanol. These stock solutions were then diluted with HPLC-grade methanol to 200, 100, 50, 25, and 12.5 μg/mL concentrations as working solutions for the quantitative calibrations. The calibration curves and quantitative evaluations were then obtained at 203 nm.

### Pharmacokinetic assessment

The quantitative determination of CK concentration in the plasma was achieved using 2 mL of intravenous blood collected from each volunteer before administration and at 2, 4, 8, and 12 h after dosing during each period. The blood samples were centrifuged at 3000 rpm for 10 min, and the supernatant was separated and frozen at -80 °C until analysis. The plasma concentrations of ginsenoside CK were determined by PCAM KOREA Co., Ltd. (Daejeon, Korea) using a HPLC–tandem mass spectrometry system. The chromatographic analysis was performed using a Waters I-class (Waters, USA), with Berberine (Dr. Ehrenstorfer GmbH, Germany) as the internal standard. Chromatographic separation was achieved with an Acquity UPLC BEH C18 column (100 mm × 2.1 mm, 1.7 μm; Waters, USA) maintained at 45 °C. The mobile phase was a gradient of 0.1% formic acid in water and 100% acetonitrile. Mass spectrometry was performed in the positive mode on an API Xevo TQ-XS instrument (Waters, USA) equipped with an electrospray ionization probe. The temperature of the ion source was set to 150 °C, and the voltage of the ion spray was 3 kV. The quantifications were performed by multiple reaction monitoring of the transitions at 645.2–203 nm for ions of ginsenoside CK, with a dwell time of 11.28 min. To validate the quantitative data in terms of linearity, the limit of detection (LOD) and limit of quantification metrics were calculated (Supplementary Table S2).

### RNA-sequencing and analysis

The total mRNA was extracted from each blood plasma sample to build the mRNA-seq libraries that were generated using a TruSeq stranded mRNA LT sample prep kit (Illumina, San Diego, CA, USA) following manufacturer protocols and sequenced using a Novaseq 6000 sequencing system (Illumina). The reads were trimmed with Trimmomatic (Bolger et al., 2014) to remove any adapters and low-quality reads, resulting in clean reads for improved paired-end mapping. The trimmed reads were mapped to the *Homo sapiens* reference genome (GRCm38) transcriptome using Salmon software version 1.3.0 (Patro et al., 2017). Differential gene expressions among the three experimental groups were evaluated using edgeR (version 3.30.3) software (McCarthy et al., 2012). The differentially expressed genes were identified based on a cutoff threshold of *p* < 0.05 and log-fold change >1 before being subjected to further analyses.

### Functional annotations

Functional annotations for each gene were made using the drug discovery protocol. The seven datasets used are included in Supplementary Table S3: DrugBank (Wishart et al., 2018), Human Protein Atlas (Uhlén et al., 2015), STITCH (Szklarczyk et al., 2016), Surfaceome (Bausch-Fluck et al., 2018), Tumor Suppressor Gene Database v2.0 (TSGene) (Zhao et al., 2016), pepBDB (Wen et al., 2019), and Comparative Toxicogenomics Database (Grondin et al., 2021). First, entered the DrugBank ID for each gene to navigate the details of known drugs from the complete database xml file. Second, downloaded the FDA-approved potential drug candidate list from the Human Protein Atlas database. Third, searched STITCH to observe small-molecule drug interactions. Fourth, used Surfaceome to understand the cell surface proteins. Fifth, used TSGene to obtain the cancer therapeutic gene candidates. Sixth, observed the peptide-binding protein interactions.

### Cell cytotoxicity assay

#### Cell cultures

Immortalized human epidermal keratinocyte (HaCaT) and murine macrophage RAW 264.7 cells were cultured in Dulbecco’s modified Eagle’s medium (DMEM) supplemented with 10% fetal bovine serum (FBS) and 1% penicillin-streptomycin. Generally, 89% Roswell Park Memorial Institute (RPMI) 1640 with 10% FBS and 1% penicillin-streptomycin were used to culture the human lung carcinoma cells (A549). All three cell lines were allowed to adhere and develop for 24 h before being treated with different samples in a humidified 37 °C incubator with a 5% CO_2_ atmosphere**.**

#### Cell cytotoxicity assay

We evaluated the cytotoxicities of DDK-401 and DDK-204 on the HaCaT and RAW 264.7 cells using an MTT colorimetric assay, which was performed in 96-well plates (Mathiyalagan et al., 2019; Pu et al., 2021). Seeding was performed at 5 × 10^4^ cells/well (HaCaT) and 1 × 10^4^ cells/well (RAW 264.7), and the 96-well plates were incubated at 37 °C in a humidified atmosphere of 5% CO_2_ for 24 h (Ramadhania et al., 2022). Subsequently, the cells were treated with various concentrations of DDK-401 or DDK-204 in serum-free-medium at 62.5, 125, 250, and 500 μg/mL for the HaCaT cells and at 25, 50, 100, 250, and 500 μg/mL for the RAW 264.7 and A549 cells, followed by incubation for 24 h. Then, 20 µL of MTT (5 mg/mL, phosphate-buffered saline (PBS), Life Technologies, Eugene, OR, USA) were added to the cells at 37 °C for 4 h. The insoluble formazan was dissolved by placing 100 μL of dimethylsiloxane (DMSO) in each well and absorbance was measured at 570 nm using an enzyme-linked immunosorbent assay (ELISA) microplate reader (Bio-Tek, Instruments, Inc., Winooski, VT, USA).

### Antioxidant assay

#### *In vitro* DPPH assay

The 2,2-diphenyl-1-picryl-hydrazyl (DPPH) method was used with a slight modification to estimate the free-radical scavenging activities of the samples (Subbiah et al., 2020). DPPH (0.2 mM) was dissolved with ethanol (pro-analysis grade) to obtain a DPPH radical solution. Then, 20 µL of the sample extract and 180 µL of the DPPH solution were added to a 96-well plate and incubated at 25 °C for 30 min in the dark, followed by absorbance measurement at 517 nm. Vitamin C (ascorbic acid) standard curves with concentrations from 0 to 100 μg/mL were used to determine the DPPH radical scavenging activity, which is expressed in milligrams of ascorbic acid equivalent per gram (mg AAE/g) of the extract.

#### Reducing power assay

The reducing capacity of a compound indicates its potential antioxidant activity. To conduct this assay (Akter et al., 2021), 100 µL of various concentrations of the samples were mixed with 250 µL of 0.2 mM phosphate buffer (pH 6.6) and 250 µL of 1% potassium ferricyanide. The mixtures were then incubated at 50 °C for 20 min. After cooling, 250 µL of 10% trichloroacetic acid was added to the mixtures and centrifuged at 3000 rpm for 10 min. Then, 50 µL of the upper layer of each mixed solution was transferred and mixed with 50 µL of distilled water and 250 µL of 0.1% ferric chloride solution in a 96-well plate. The absorbance was then measured at 700 nm using a UV spectrometer microplate reader (Bio-Tek, Instruments, Inc., Winooski, VT, USA). Vitamin C was used as the standard, and a blank solution was prepared by omitting the sample; the results are expressed as mg AAE/g of extract.

#### Reactive oxygen species generation assays in HaCaT and lung cancer cells

##### Effects of DDK-401 on reactive oxygen species production in HaCaT cells under oxidative stress

Intracellular reactive oxygen species (ROS) were determined using the 2′,7′-dichlorodihydro-fluorescein diacetate (DCFH-DA) reagent, as described by Pu et al. (2021), with a slight modification. Briefly, HaCaT cells (5 × 10^4^ cells/well) were seeded in a 96-well plate (Nest Inc., Corning, NY, USA) and incubated for 24 h at 37 °C and 5% CO_2_. To assess the antioxidant activity, the cells were treated with H_2_O_2_ (500 μmol/L) for 2 h, and the supernatant was aspirated. The cells were then either treated or not treated with different concentrations of the samples for 24 h. Vitamin C was used as the positive control. After washing the cells twice with PBS, we added 20 μM DCFH-DA in PBS and incubated them for another 20 min. The supernatant was next removed by washing the cells with PBS twice, and a multimodal plate reader was used to measure the fluorescence intensity at an excitation wavelength of 485 nm and emission wavelength of 528 nm.

##### Effects of DDK-401 on reactive oxygen species production in A549 cells under oxidative stress

To detect the ROS intensity of human lung cancer cells (A549), we used the DCFH-DA reagent with fluorescent image capture technique. We plated the cells at a density of 1 × 10^4^ cells/well in 96-well culture plates, allowed them to adhere, and then placed them in an incubator overnight to achieve 100% confluency. The A549 cells were then treated with various concentrations of DDK-401 or DDK-204 (0, 25, 50, 100, 250, and 500 μg/mL) for 24 h. The next day, the cells were stained by adding 100 μL of DCFH-DA solution (10 μM) to each well and incubated in the dark for 30 min. The old media were discarded, and the cells were washed twice with 1× PBS (100 μL/well). A multimodal plate reader (spectrofluorometer) was used to determine the fluorescence intensity caused by ROS production at an excitation wavelength of 485 nm and emission wavelength of 528 nm.

### Inflammation inhibition assay

The detection of nitric oxide (NO) levels has been described previously (Ramadhania et al., 2022). The RAW 264.7 cells (1 × 10^4^) were placed in 24-well culture plates and incubated for 24 h at 37 °C in a humidified environment with 5% CO_2_. Then, they were treated with different concentrations of DDK-401 or DDK-204 (0, 25, 50, 100, 250, and 500 μg/mL) for 1 h. In the presence of the samples, 1 μg/mL lipopolysaccharide (LPS) was used as the stimulator, and the treated cells were placed in an incubator for one day. The nitrite levels in the cell media were determined using the Griess reagent: 100 μL of the stimulated supernatant was mixed with an equivalent volume of the Griess reagent. A microplate reader was used to compare the absorbance at 540 nm with a standard curve obtained using sodium nitrite (BioTek Instruments, Inc.). L-NMMA (50 μM), a standard inhibitor, was used as the positive control in this experiment. Each assay was repeated three times, and the results are expressed in terms of percentage of NO production.

### Reverse transcription polymerase chain reaction (RT-PCR)

The total RNA was extracted using QIAzol lysis reagents (QIAGEN, Germantown, MD, USA), and the reverse transcription reactions were performed using 1 µg of total RNA in 20 µL of the reaction buffer with an amfiRivert reverse transcription kit (GenDepot, Barker, TX, USA), according to manufacturer instructions. The obtained cDNA was amplified with primers, as shown in Table 1. The reaction was cycled 35 times: 30 s at 95 °C, 30 s at 60 °C, and 50 s at 72 °C. Using 1% agarose gels, the amplified RT-PCR products were analyzed, visualized using Safe-Pinky DNA Gel Staining (GenDepot, Barker, TX, USA), and imaged under ultraviolet light.

**TABLE 1:** List of primers and their sequences used for mRNA gene expression validation by RT-PCR.

Gene	Primer sequence (5′-3′)
FOXO3	F: TCA AGG ATA AGG GCG ACA GC
	R: GGA CCC GCA TGA ATC GAC TA
TLR4	F: GAG GAC TGG GTG AGA AAC GA
	R: GAA ACT GCC ATG TCT GAG CA
Caspase 8	F: AGA GTC TGT GCC CAA ATC AAC
	R: GCT GCT TCT CTC TTT GCT GAA
MMP 9	F: CGT CGT GAT CCC CAC TTA CT
	R: AGA GTA CTG CTT GCC CAG GA
p38	F: CGA CTT GCT GCT GGA GAA GAT GC
	R: TCC ATC TCT TCT TGG TCA AGG
GAPDH	F: CAA GGT CAT CCA TGA CAA CTT TG
	R: GTC CAC CAC CCT GTT GCT GTA G

### Statistical analysis

All experiments were performed at least in triplicate (*n* = 3) unless stated otherwise. The experimental data are reported as mean ± standard error (SEM). Statistical significances between the control and sample groups were evaluated by Student’s t-test with a two-tailed distribution and two-sample equal variances. A greater extent of statistical significance is indicated by an increasing number of asterisks (*p < 0.05, **p < 0.01, and ***p < 0.001) and hash markers (#*p* < 0.05, ##*p* < 0.01 and ###*p* < 0.001). The hash marker (#) indicates significance between the normal and stimulated controls, and the asterisk (*) indicates significant differences between the stimulation groups (DDK-204 or DDK-401).

## Results and discussion

### Ginsenoside absorption profiling after oral intake

#### Preparation of DDK-401 and DDK-204

Ginsenosides and ginseng extracts have been reported to have various pharmacological effects, and ginseng has been used as a medicinal herb in TCM for several centuries. However, ginsenosides are mainly absorbed in the gastrointestinal tract after the gut microbes hydrolyze the linear carbohydrates from their backbones. In addition, the minor ginsenosides, which have only one or no glycose moieties, generally reach the systemic circulation. Thus, the absorption and bioavailability of ginsenosides greatly depend on the gastrointestinal bioconversion ability of each individual, and the minor saponins must be enriched by various processing technologies. Bioconversion techniques such as puffing (Pu et al., 2021) and heat treatment (steaming) do not produce ginsenoside CK (Piao et al., 2020), which is one of the active metabolites that reaches systemic circulation and has various pharmacological activities (Sharma and Lee, 2020). Therefore, the pharmacologically active minor saponin CK must be enriched by the edible enzymes in the ginseng extract to maximize its biological activity irrespective of an individual’s gut function. This study, we aimed to increase the total ginsenoside and CK content using pectinase and *β*-glucosidase enzymes to begin the glucose hydrolysis of major ginsenosides in red ginseng concentrate, such as Rb1, Rd, and Rg3. We performed additional fermentation with *Lactobacillus* species at 37 °C for 1 day to accelerate the hydrolysis of the glucose molecules from the major ginsenosides to increase CK production (Table 2). The synthesis of CK has been mainly reported from the hydrolysis of glycose molecules of the major ginsenosides, such as Rb1, Rb2, Rd, Rc, compound O, compound Y, compound Mc, Rg3, gypenoside XVII, and F2 (Sharma and Lee, 2020). As a result of the above processes, the fermented red ginseng extract (DDK-401) was enriched, with 10 mg/g of CK and 32.98 mg/g of total ginsenoside content, compared to the control red ginseng extract (DDK-204), which contained 9.72 mg/g of total ginsenosides and without CK. The CK was thus clearly produced by the fermentation process and not the steaming process (Piao et al., 2020). It was previously reported that the bioconversion and fermentation of red ginseng yields CK in Korean ginseng (Choi et al., 2016; Fukami et al., 2018).

**TABLE 2:** Ginsenoside profiles of DDK-401 and DDK-204 for *in vivo* pharmacokinetic assessments.

Samples	Rg1	Re	Rf	Rb1	Rg2	Rc	Rb2	Rb3	Rd	F2	Rg3	Rk1	Rg5	CK	Total (mg/g)
DDK-401 Fermented Ginseng Extract	1.66 ± 0.035	2.77 ± 0.025	0.38 ± 0.046	5.26 ± 0.123	2.35 ± 0.059	3.08 ± 0.061	3.6 ± 0.095	0.22 ± 0.010	1.05 ± 0.055	0.00 ± 0.000	0.78 ± 0.026	0.63 ± 0.080	0.5 ± 0.025	**10.69 ± 0.040**	32.98 ± 0.284
DDK-204 Red Ginseng Extract	0.96 ± 0.026	1.41 ± 0.031	0.39 ± 0.020	2.87 ± 0.051	0.22 ± 0.005	1.46 ± 0.026	1.17 ± 0.049	0.15 ± 0.040	0.57 ± 0.055	0.00 ± 0.000	0.22 ± 0.031	0.17 ± 0.021	0.15 ± 0.040	**0.00 ± 0.000**	9.72 ± 0.208

#### Ginsenoside absorption profiling after oral intake of DDK-401

**FIGURE 2:**
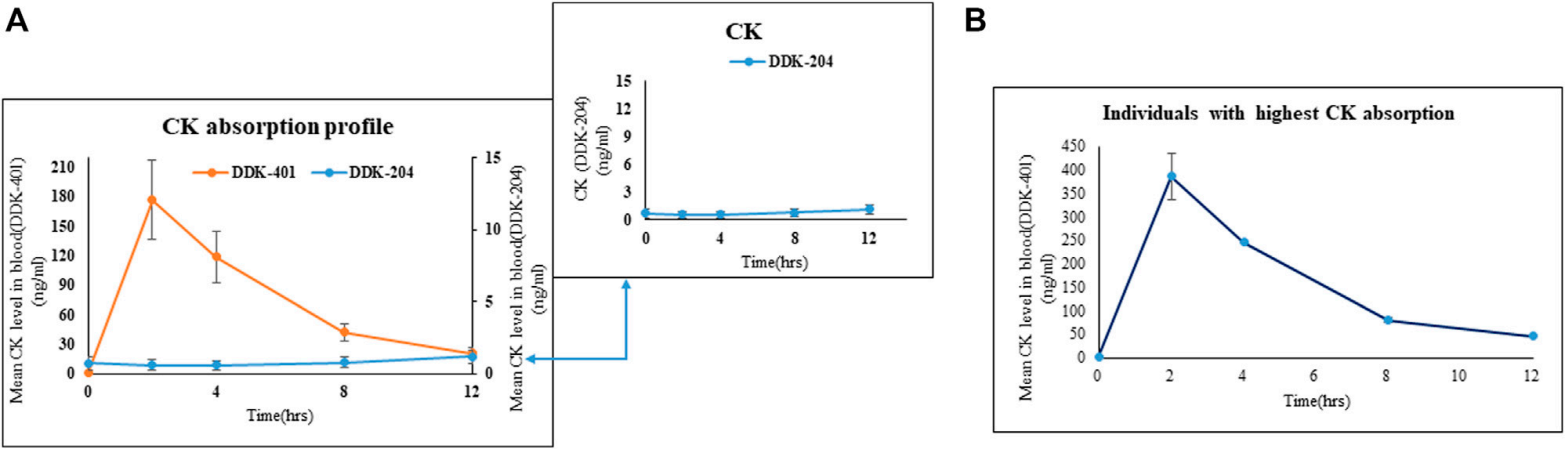
Absorption profiles of CK in human blood after oral intake of fermented red ginseng extract (DDK-401) and red ginseng extract (DDK-204). **(A)** Mean CK level. Inset shows the mean CK absorption profile after DDK-204 intake. **(B)** Mean CK level from individuals with highest CK absorption in this study.

After oral intake, the human volunteers showed greater CK absorption from the fermented DDK-401 extract than from the control red ginseng extract (DDK-204) (Figure 2A). The T_max_ was 2.4 h, C_max_ was 184.8 ± 39.64 ng/mL, and AUC_0–12h_ was 920.3 ± 194.70 ng∙h/mL for DDK-401, whereas the T_max_ was 12 h, C_max_ was 2.5 ± 1.09 ng/mL, and AUC_0–12h_ was 11.3 ± 4.66 ng∙h/mL for DDK-204 (Table 3). These pharmacokinetic patterns are similar to those in other reports (Sharma and Lee, 2020). Although various studies have reported enhanced CK absorption after oral administration of fermented red ginseng extract, the concentration of CK in the blood plasma still varies by individual, as shown in Figure 2B (individuals with the highest CK absorption profiles).

**TABLE 3:** Pharmacokinetic parameters of CK in human blood after oral intake of fermented ginseng extract (DDK-401) and red ginseng extract (DDK-204).

Parameters	DDK-401	DDK-204
T_max_ (h)	2.4 ± 0.27	12.0 ± 0.00
C_max_ (ng/mL)	184.8 ± 39.64	2.5 ± 1.09
AUC_0-12h_ (ng·h/mL)	920.3 ± 194.70	11.3 ± 4.66

#### Variations in the CK absorption profiles between male and female groups

**FIGURE 3:**
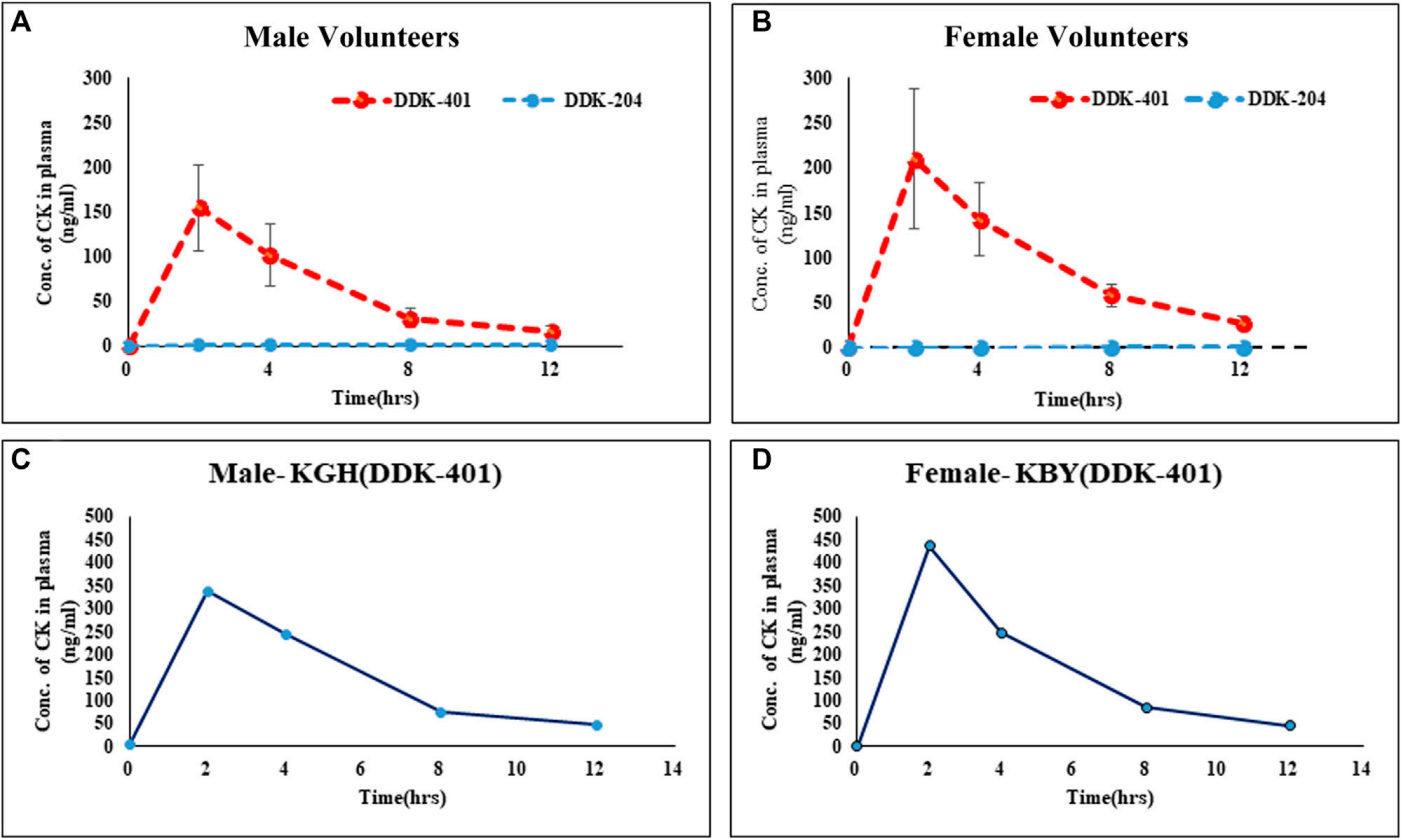
Absorption profiles of CK in human blood serum after oral intake of fermented red ginseng extract (DDK-401) and red ginseng extract (DDK-204): **(A)** male volunteers, **(B)** female volunteers; maximum CK absorption in individual **(C)** male and **(D)** female.

Although high CK absorption has been reported previously (Sharma and Lee, 2020), differences in the absorption patterns between males and females following oral intake of fermented red ginseng extract have not been explored. Our results indicate that as a group, the female volunteers absorbed more CK (Figure 3B) than the male volunteers (Figure 3A), although this pattern also applied to individual female and male volunteers (Figure 3C). Similarly, the female volunteers were previously reported to absorb higher concentrations of CK than males after oral doses of a high concentration of CK (Chen et al., 2017).

### RNA sequence analysis of blood plasma after oral intake of samples

#### Differential gene expression and KEGG pathway enrichment

**FIGURE 4:**
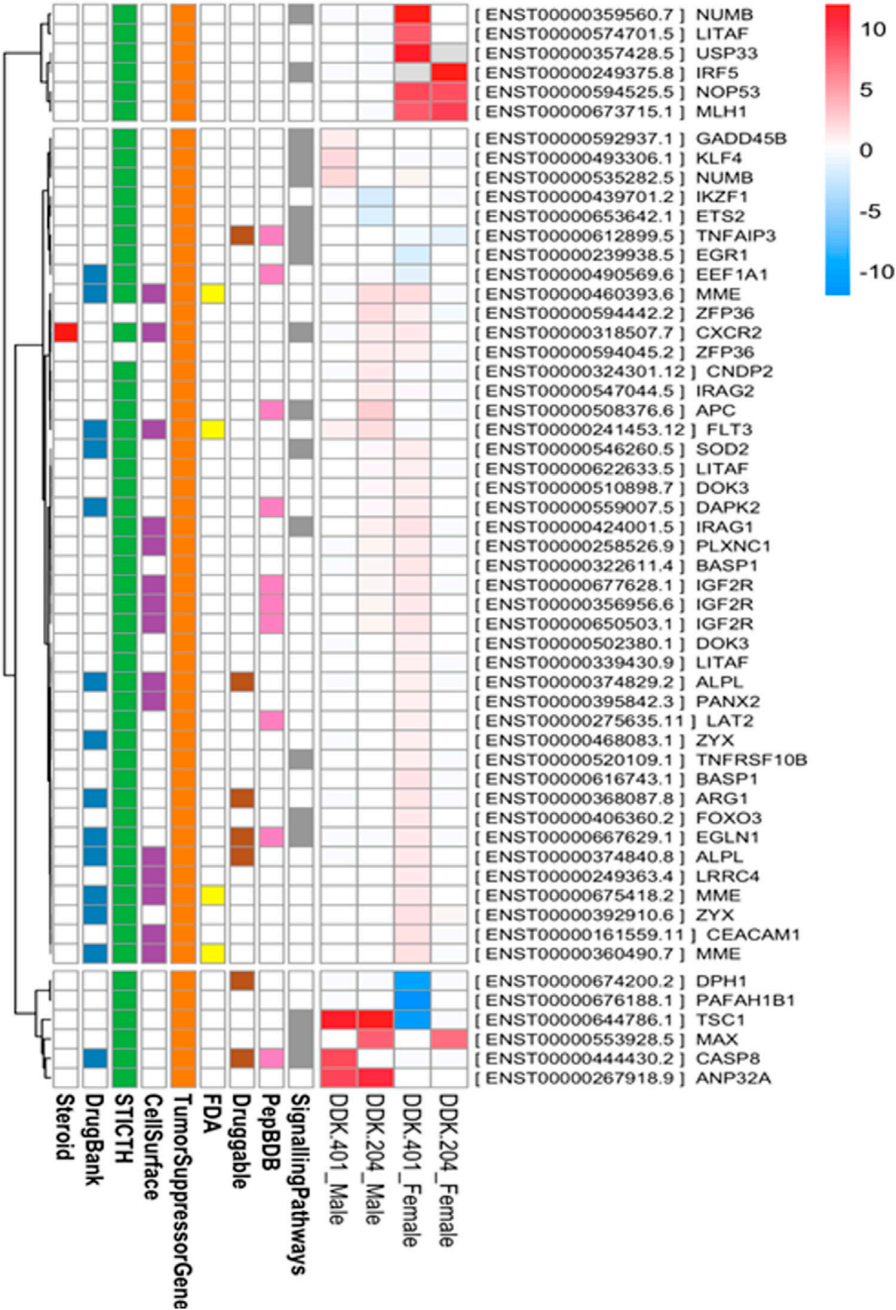
Transcripts belonging to the “tumor suppressor genes” category that were differentially expressed between the DDK-401 male and female groups and DDK-204 male and female groups displayed as a heatmap.

As explained in the *Pharmacokinetic Assessment* section, whole mRNA transcripts were assessed against the human reference genome for genome-wide differential transcript expressions. The samples were grouped into four categories, namely DDK-401 (male and female) and DDK-204 (male and female). Overall, 701 transcripts were found to have differential expressions (Supplementary Table S3), and the transcripts overlapped among the groups, as illustrated in a Venn diagram (Supplementary Figure S1). The transcripts belonging to the tumor suppressor genes category are displayed in a heatmap (Figure 4). Overall, nine annotations were included in this study, as explained earlier. In addition, all differentially expressed genes were subjected to KEGG pathway enrichment in the David online webserver, which showed that the cell death pathways, such as apoptosis and necroptosis, were enriched by the extract treatments (Supplementary Table S4). Finally, we selected gene candidates (FOXO3, cysteine-aspartic protease 8 (caspase-8), toll-like receptor 4 (TLR4), and matrix metallopeptidase 9 (MMP-9)) for the RT-PCR expression analysis because these are known to be involved in the signaling and cell-death pathways as well as tumor suppression.

### Effects of DDK-401 on the viabilities of HaCaT and lung cancer cells

**FIGURE 5:**
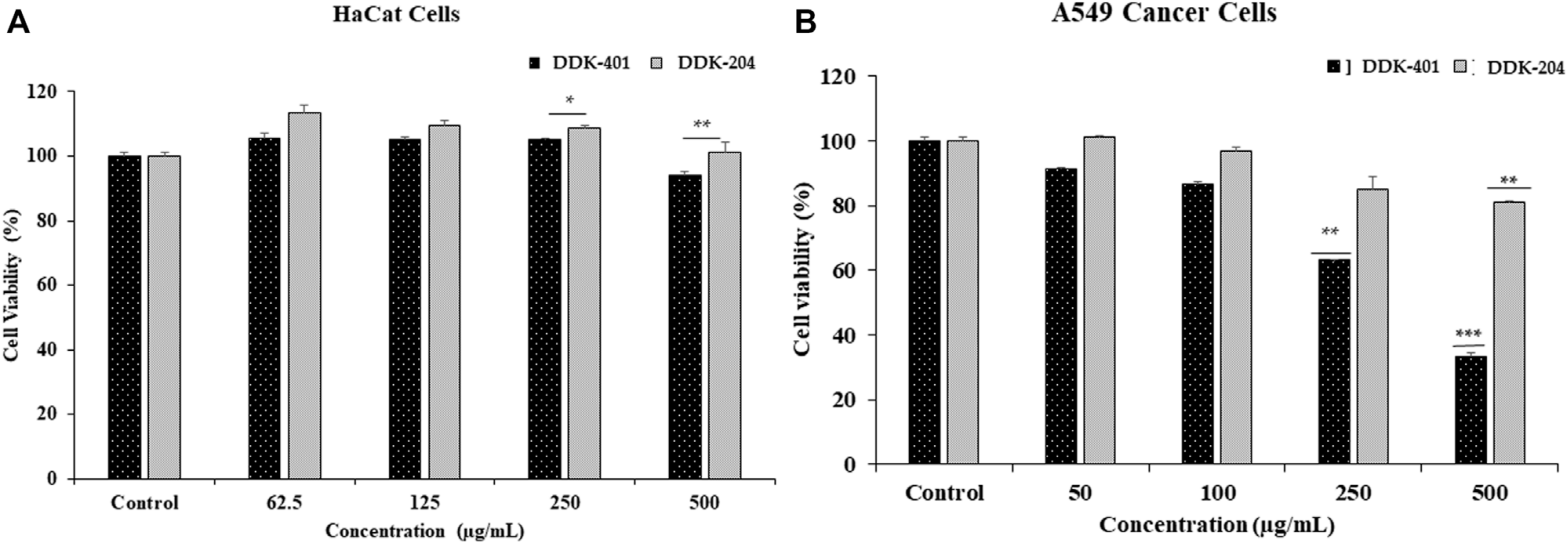
*In vitro* cytotoxicities of DDK-401 and DDK-204 samples in **(A)** HaCaT and **(B)** A549 lung cancer cells over 24 h. The graph shows the mean ± SD value of four repetitions. **p < 0.01; ***p < 0.001 indicate significant differences from the control groups.

The cytotoxicities of DDK-401 and DDK-204 to HaCaT cells was determined for safety purposes. The HaCaT cells represent normal cell conditions, and lung cancer cells (A549) were used to examine the apoptosis signaling pathway. Each sample was evaluated at various sample concentrations (62.5, 125, 250, and 500 μg/mL in HaCaT and 25, 50, 100, 250, and 500 μg/mL in A549 cells). As shown in Figure 5, at concentrations less than 500 μg/mL, both DDK-401 and DDK-204 were nontoxic to HaCaT cells. In the A549 cells, DDK-401 demonstrated minimal toxicity after 24 h at 250 μg/mL. At a concentration of 500 μg/mL after 24 h, DDK-401 showed significantly decreased cancer cell proliferation than DDK-204. Moreover, A549 cell viability was reduced by DDK-401 in a dose-dependent manner. The cytotoxicity results in this investigation match those in a previous report (Yu et al., 2018).

The results shown in Figure 5 indicate that at 500 μg/mL, DDK-401 and DDK-204 were only mildly toxic, from which it can be concluded that both substances are relatively safe when cell conditions are normal. On the other hand, in lung cancer A549 cells, which represent cell damage and imbalanced conditions, DDK-401 had a much higher toxicity than DDK-204, producing apoptosis of the cancer cells.

### Antioxidant content shown by DPPH assay and ROS generation in HaCaT and cancer cells

#### Antioxidant capacity: DPPH and reducing power assays

The DPPH scavenging and potassium ferricyanide reducing power assays were used to evaluate the antioxidant capacities of DDK-401 and DDK-204, and the results are shown in Table 4. The most frequently used antioxidant standard for these assays is vitamin C; therefore, the results of the DPPH and potassium ferricyanide reducing power assays are expressed in terms of mg AAE/g of the extract. These assays are widely used to determine the antioxidant properties of compounds as free radical scavengers or hydrogen donors (Warinhomhoun et al., 2021) as well as the ability of the compounds to transform from Fe^3+^/ferricyanide complex to Fe^2+^/ferrous forms (Aryal et al., 2019). DDK-401 showed higher antioxidant abilities in both the DPPH and potassium ferricyanide reducing power assays, with values of 0.093 ± 0.02 and 0.340 ± 0.001 mg AAE/g of extract, respectively. In the DPPH assay, the antioxidant capacity of DDK-401 was generally 2 times higher than that of DDK-204, and in the potassium ferricyanide reducing power assay, it was 3 times higher than that of DDK-204. In agreement with a previous study (Jung et al., 2019; Park et al., 2021), we found that CK-enriched ginseng extract (DDK-401) exhibited greater antioxidant activity than the common red ginseng extract (DDK-204). This result could be attributed to CK’s potential for radical scavenging activity in antioxidant assays (Baik et al., 2021). Antioxidants, whether endogenously produced or supplied by external sources, can scavenge ROS and reduce cellular oxidation, thereby alleviating oxidative stress (Liu et al., 2018).

**TABLE 4:** Antioxidant capacity of DDK-401 and DDK-204.

Sample	DPPH	Reducing power
	(mg AAE^a^/g extract)	(mg AAE^a^/g extract)
DDK-401	0.093 ± 0.02	0.340 ± 0.001
DDK-204	0.049 ± 0.01	0.097 ± 0.002

^a^ mg AAE/g extract: mg ascorbic acid equivalents/g extract; DPPH: 2,2-diphenyl-1-picrylhydrazyl radical scavenging assay.

#### Effect of DDK-401 on ROS production in HaCaT cells with H_2_O_2_-induced oxidative stress

**FIGURE 6:**
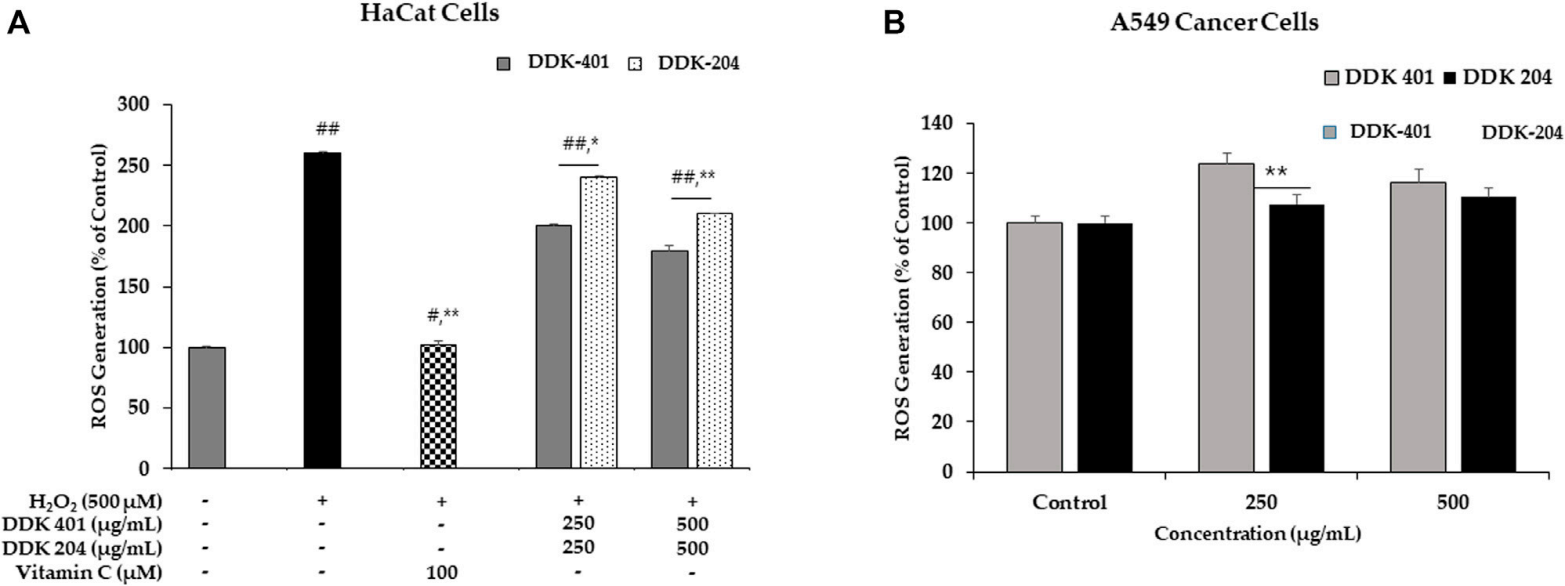
**(A)** Reactive oxygen species (ROS) inhibition by DDK-401 and DDK-204 in HaCaT cell lines treated with H_2_O_2_. The graph shows the mean ± SD value of three repetitions. *p < 0.05 and **p < 0.01 indicate significant differences from the control groups; #*p* < 0.05 and ##*p* < 0.01 indicate significant differences from the H_2_O_2_ stimulation groups. **(B)** ROS generation by DDK-401 and DDK-204 in A549 cancer cells. The graph shows the mean ± SD value of three repetitions. **p < 0.01 indicates significant difference from the control group.

We used the DCFH-DA assay to investigate the antioxidant properties of DDK-401 and determine whether it could reduce accumulated intracellular ROS in H_2_O_2_-induced HaCaT cells. Commonly, H_2_O_2_ is used to induce intracellular ROS and produce imbalance in the cellular oxidant–antioxidant levels. Because the mitochondria are the major sources of ROS, mitochondrial dysfunction caused by excess ROS can lead to apoptosis and DNA damage (Zhang et al., 2020). The mean value of the ROS levels measured in the group treated with 500 μM H_2_O_2_ was 260% higher than that in the control group. The trend of decreased cell viability after H_2_O_2_ exposure is shown in Figure 6A. Vitamin C was used as the positive control. For H_2_O_2_-induced oxidative stress in the HaCaT cells, DDK-401 was stronger than DDK-204 in a dose-dependent manner. At a concentration of 250 μg/mL, DDK-401 and DDK-204 reduced ROS levels by an average of 23% and 7%, respectively, compared with the group treated with only H_2_O_2_ (Figure 6A). These results may be attributed to the CK in DDK-401; previous studies have reported that CK activates the NF-κB and JNK pathways, which contribute to the inhibition of TNF-α and anti-inflammatory activity related to ROS inhibition (Choi et al., 2007; Park et al., 2012). Oxidative stress usually activates certain signaling pathways, including the p38, MMP, and caspase pathways.

#### Effect of DDK-401 on ROS generation to induce apoptosis of cancer cells

In A549 cells, the DCFH-DA reagent was used to measure the intracellular ROS levels with DDK-401 and DDK-204 at various concentrations. Red ginseng extract has been shown to induce cancer cell death by causing DNA damage, stimulating ROS production, and activating numerous pro-apoptotic markers. Furthermore, mitochondrial damage can cause release of ROS because the mitochondria are the largest source of ROS (Brunelle and Chandel, 2002). At 500 g/mL, DDK-401 produced a higher level of ROS than DDK-204, as shown in Figure 6B. Thus, the antiproliferative action of DDK-401 could be assessed by measuring the ROS levels. Intracellular oxidative stress is known to cause cell death in a variety of cell lines; therefore, this assessment was crucial. The data show that DDK-401 could be a potential drug candidate in clinical trials for the treatment of lung cancer.

ROS have been identified as signaling molecules in various pathways that regulate both cell survival and cell death depending on the level (Azad et al., 2008; Chen et al., 2018). Apoptosis and autophagy are important molecular processes that maintain balance in organisms and cells. Apoptosis destroys damaged or unwanted cells, while autophagy maintains cellular homeostasis by recycling specific intracellular organelles and molecules, although autophagy can result in cell death in some cases (Thorburn, 2008; Fan and Zong, 2013). We conclude that DDK-401 reduced ROS production in normal cells (HaCaT) experiencing oxidative stress and also led to apoptosis of lung cancer (A549) cells, suggesting that DDK-401 could act as a molecular switch for these two cellular processes in response to cell damage signaling.

### Effect of DDK-401 on gene expression affecting apoptotic and inflammatory responses

**FIGURE 7:**
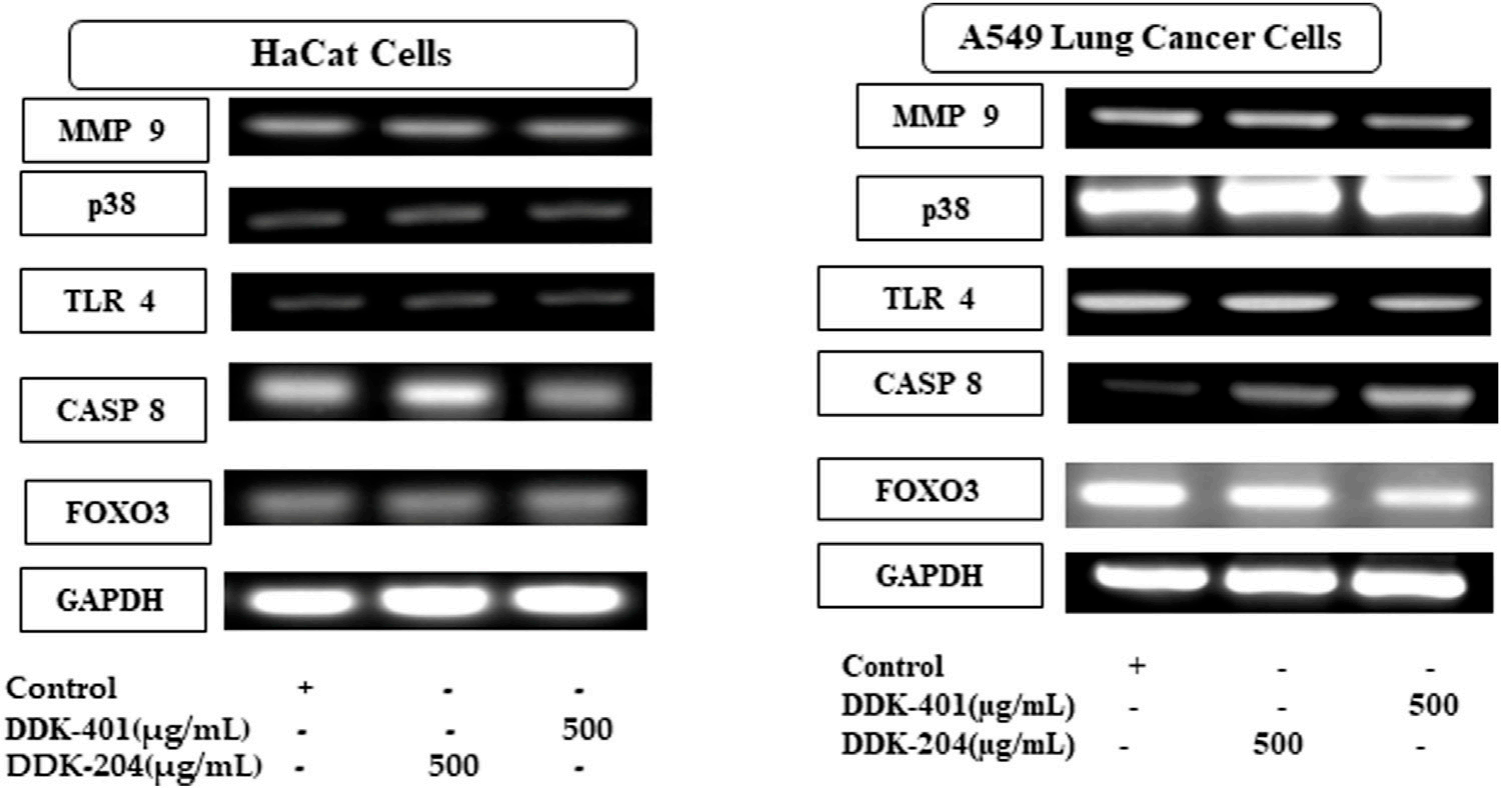
*In vitro* validation of the selected candidate gene expressions in normal (HaCaT) and lung cancer (A549) cell lines by RT-PCR.

Living cells produce ROS as a normal metabolic byproduct. Under excessive stress, the cells generate excess ROS, so living organisms have evolved a series of response mechanisms to adapt to ROS exposure and use ROS as a signaling molecule. ROS molecules cause oxidative stress in a feedback mechanism involving numerous biological processes, including apoptosis, necrosis, and autophagy (He et al., 2017). Apoptosis is a normal process that occurs during development and aging as well as functions as a homeostatic mechanism to maintain cell populations in tissues. Apoptosis can even occur as a defense mechanism during immune responses or when cells are damaged by disease or toxins (Elmore, 2007). In this study, we investigated several gene markers that we selected through a whole-transcriptome search for differential expressions. We found differentially expressed genes related to apoptosis and immune responses to inflammation, such as FOXO3, TLR4, caspase-8, MMP-9, and p38 MAP kinase (p38) (Cuadrado and Nebreda, 2010; van der Vos and Coffer, 2011; Zheng et al., 2021). In addition, we investigated the effects of DDK-401 and DDK-204 without any stimulation (UV-B irradiation) in HaCaT cells to observe whether our samples could trigger inappropriate apoptosis or inflammation under normal conditions showed in (Figure 7). The RT-PCR analysis (Supplementary Figure S3) showed that in HaCaT cells, neither compound regulated FOXO3, TLR4, MMP-9, or p38 expression, indicating that DDK-401 did not trigger inappropriate apoptosis or inflammation under normal conditions. The ability to control cellular living or death has enormous therapeutic potential. However, upregulation of caspase-8 was observed, which is similar to the RNA-seq data indicating differential expression (Figure 4). Although the activation of caspase-8 is mainly associated with death receptor signaling cascades, it is also activated downstream of the mitochondria. The roles of caspase-8 in the shift from autophagy to apoptosis in cisplatin-resistant MCF7 cells and in TRAIL-mediated autophagy in HCT 116 cells have already been studied (de Vries et al., 2006; Hou et al., 2010). In the lung cancer A549 cells, DDK-401 treatment downregulated the expressions of MMP9 and TLR4 while upregulating the expressions of p38 and caspase-8 genes, compared with the cells treated with DDK-204. The MMPs are a group of zinc-dependent metalloenzymes that regulate various cellular processes, including tumor cell proliferation and metastasis (Guo et al., 2019). Several studies have noted that MMPs are overexpressed in malignant tissues than the adjacent normal tissues in a range of tumors, including lung, colon, breast, and pancreatic carcinomas (Radad et al., 2011; Nakhjavani et al., 2019). Previous research has shown that downregulating the expressions of intracellular MMP-9 can increase invasion and metastasis processes several cancers (Zhang et al., 2017, Liu et al., 2019). Furthermore, oxidative stress can induce receptor-dependent apoptosis and damage the mitochondria of normal cells. Mitochondrial dysfunction then further increases ROS accumulation and activates the p38 MAPK pathway. ROS can continuously activate p38 MAPK by activating MAPK kinase and inhibiting MAPK phosphatase. In A549 cells, ROS can regulate the expressions of Bax and Bcl-2 by activating p38 MAPK, which increases the level of cytochrome c in the cytoplasm and triggers the caspase cascade reaction leading to apoptosis (Jin et al., 2019; Nguyen and Nguyen, 2019). The caspases are a family of cysteine-containing proteolytic enzymes that play a central role in the execution phase of cell apoptosis. It has been reported that the effects of the caspase 8 pathway on cancer cells involve inducing apoptosis (Huang et al., 2019). The apoptosis induced by most anticancer drugs occur by the activation of caspases (González-Burgos et al., 2015; Kim and Kim, 2018). Caspase-8 is important in the death receptor-mediated extrinsic pathway, and DDK-401 promotes the activation of caspase-8 in A549 cells in this study, showing that it can cause apoptosis by activating the extrinsic caspase pathway (Wu et al., 2019; Yi, 2019). TLR4 is an important member of the type I transmembrane protein family. Recently, growing evidence has shown TLR4 in various tumors (Gao et al., 2017; Li and Ji, 2018; Zhou et al., 2019), including head and neck, lung, gastrointestinal, liver, pancreatic, skin, breast, ovarian, cervical, and prostate cancers. TLR4-mediated cancer growth is involved in breast tumor progression, and the downregulation of TLR4 prevented breast cancer progression and improved survival (Zhou et al., 2019c). According to our findings, DDK-401 could induce cell apoptosis by upregulating and downregulating various transcriptional factors under cancerous conditions.

## Conclusion

Although ginseng and ginsenosides have been reported to have various pharmacological effects, the uptake of ginsenosides into systemic circulation, which is required for their effectiveness, depends on individual factors. Because CK was reported to be a minor saponin that reached systemic circulation, we enriched the total ginsenoside and CK content by fermenting red ginseng extract (DDK-401) via bioconversion and fermentation by edible enzymes. Because clinical trials are a prompt option for evaluating product efficacy, we evaluated DDK-401 in a clinical trial of healthy Korean volunteers. We found higher CK in blood plasma after oral intake of DDK-401 than after the consumption of the control red ginseng formula. Moreover, we identified differences in the CK absorption patterns between female and male volunteers, with higher concentrations of CK being detected in females than in males. We also observed differential expression patterns of various tumor suppressor genes between the female and male groups through RNA-seq analysis. DDK-401 exhibited no cytotoxicity in normal non-diseased HaCaT and RAW 264.7 cells, whereas it showed cytotoxicity in lung cancer cells (A549). Furthermore, DDK-401 inhibited H_2_0_2_-induced ROS production in HaCaT cells and increased ROS production in cancer cells. Finally, the candidate genes responsible for apoptosis and inflammation were validated using RT-PCR (Figure 7). This is a pilot study reporting that fermented red ginseng extract (DDK-401) produces unique, differential absorption and gene regulation patterns compared with red ginseng extract (DDK-204). Thus, DDK-401 could be a potential candidate for further investigations in clinical trials for health promoting activities and anticancer agents; various nanoformulations could also be considered to boost its bioavailability and anticancer properties.

## Data Availability

The datasets presented in this study can be found in online repositories. The names of the repository/repositories and accession number(s) can be found below: ncbi.nlm.nih.gov, PRJNA873242.
