# Supplementary material for: Transcriptome expression profile of compound-K-enriched red ginseng extract (DDK-401) in Korean volunteers and its apoptotic properties
Source: Front Pharmacol. 2022 Dec 1;13:999192. doi: 10.3389/fphar.2022.999192 (PMC9751427; doi:10.3389/fphar.2022.999192)
Supplement: Supplementary file 2 [file Table1.docx]

**Supplementary Table 1.**  The patient age information used in this study.

| **PATIENT** | **GENDER** | **AGE** |
| --- | --- | --- |
| YJE | Female | 26 |
| ZMJ | Female | 20 |
| SJH | Female | 23 |
| KBY | Female | 25 |
| JMH | Male | 27 |
| CMH | Male | 19 |
| KTG | Male | 25 |
| KGH | Male | 20 |
| LSJ | Male | 20 |
| CHS | Male | 19 |
| KHW | Male | 25 |
| GDH | Male | 25 |
