# Supplementary figures and images for: Transcriptome expression profile of compound-K-enriched red ginseng extract (DDK-401) in Korean volunteers and its apoptotic properties
Source: Front Pharmacol. 2022 Dec 1;13:999192. doi: 10.3389/fphar.2022.999192 (PMC9751427; doi:10.3389/fphar.2022.999192)

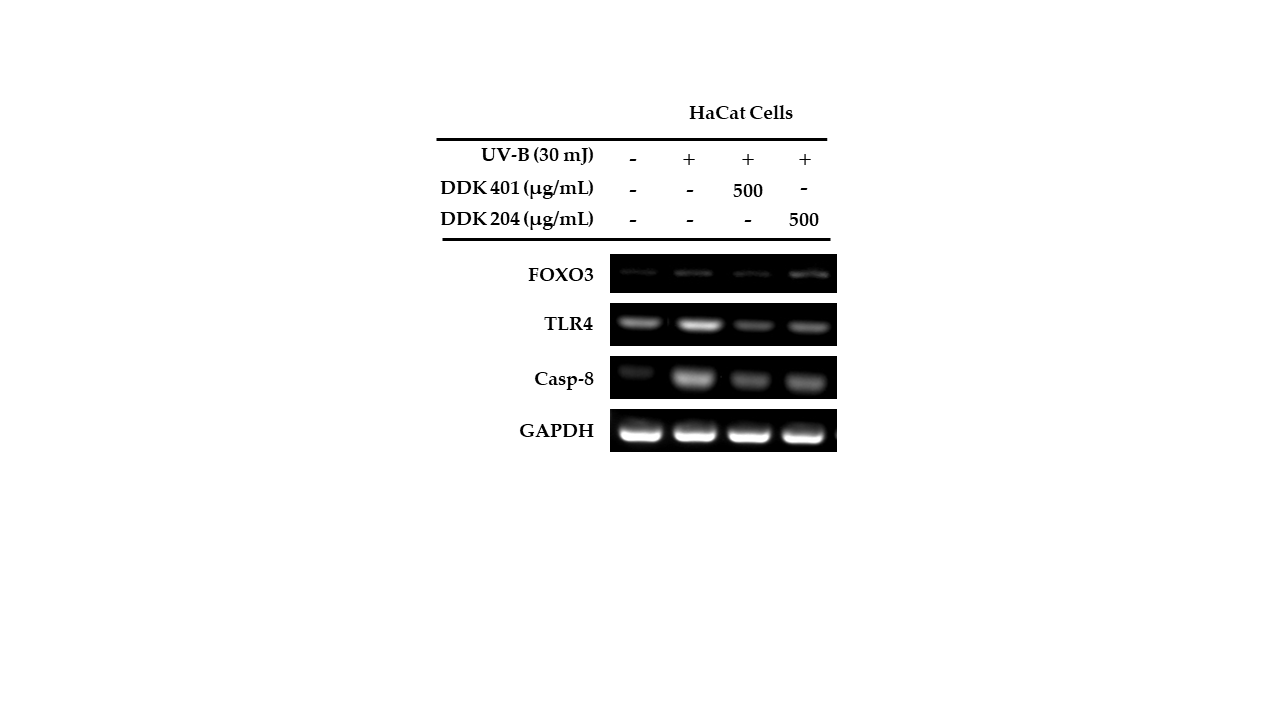

Supplement: Supplementary file 3 [file Image3.tif]

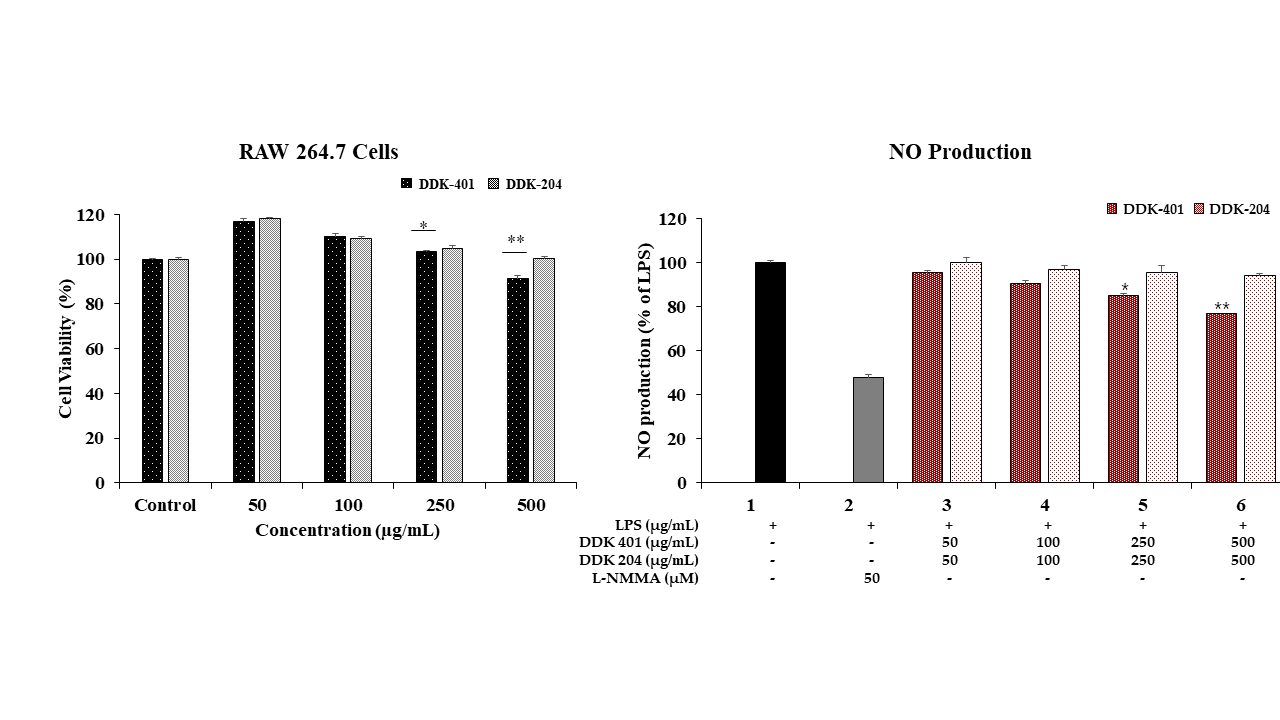

Supplement: Supplementary file 4 [file Image2.tif]

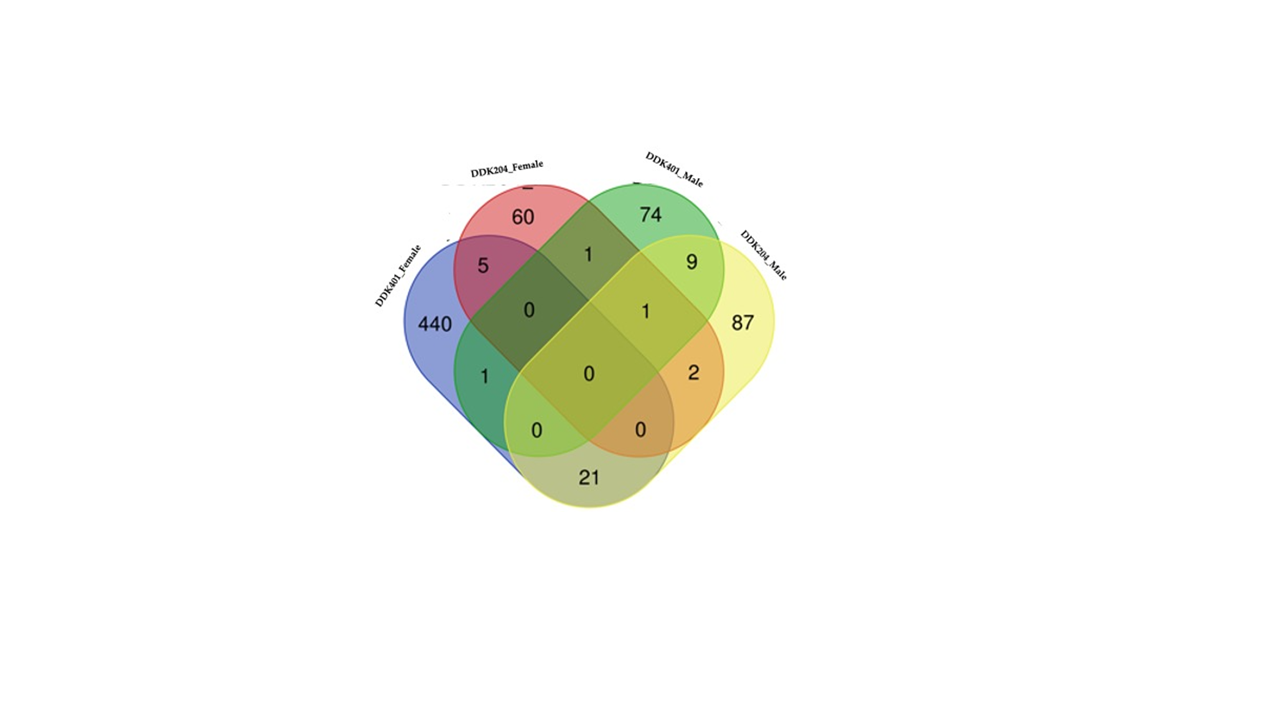

Supplement: Supplementary file 5 [file Image1.tif]
