# Supplementary material for: Transcriptome expression profile of compound-K-enriched red ginseng extract (DDK-401) in Korean volunteers and its apoptotic properties
Source: Front Pharmacol. 2022 Dec 1;13:999192. doi: 10.3389/fphar.2022.999192 (PMC9751427; doi:10.3389/fphar.2022.999192)
Supplement: Supplementary file 6 [file Table2.docx]

**Supplementary Table 2.** Parameter of Calibration curves, LOD and LOQ of reference standard compounds

| **Standard** | **Solvent** | **Regression equation y=ax+b** | **R^2^** | **LOD (mg/L)** | **LOQ (mg/L)** |
| --- | --- | --- | --- | --- | --- |
| Rb1 | Methanol | y=0.00295412x+0.000149945 | 0.998862 | 0.03 | 0.09 |
| CK |  | y=0.016227x+0.000731736 | 0.999968 | 0.034333 | 0.103 |
